# 3-Methyl-1,2,3,4,5,6,1′,2′,3′,4′-deca­hydro­spiro­[benz[*f*]isoquinoline-1,2′-naphthalen]-1′-one

**DOI:** 10.1107/S1600536812043309

**Published:** 2012-10-27

**Authors:** Sohro Siaka, Anatoly T. Soldatenkov, Anastasia V. Malkova, Elena A. Sorokina, Victor N. Khrustalev

**Affiliations:** a Institut National Polytechnique Félix HOUPHOUËT–BOIGNY, BP 1093, Yamoussoukro, Côte d’Ivoire; b Organic Chemistry Department, Peoples’ Friendship University of Russia, Miklukho–Maklaya St 6, Moscow, 117198, Russia; c X-Ray Structural Centre, A.N. Nesmeyanov Institute of Organoelement Compounds, Russian Academy of Sciences, 28 Vavilov St, B-334, Moscow 119991, Russian Federation

## Abstract

The title compound, C_23_H_23_NO, is the product of a tandem transformation of the double Mannich base bis­(1-oxo-1,2,3,4-tertrahydro-2-naphtho­ylmeth­yl)amine hydro­chloride in HBr solution upon heating. The tetra­hydro­pyridine ring has a non-symmetrical half-chair conformation, whereas the cyclo­hexa­diene and cyclo­hexene rings adopt non-symmetrical half-boat conformations. The dihedral angle between the planes of the terminal benzene rings is 62.85 (6)°. The N atom has a trigonal–pyramidal geometry [sum of the bond angles = 332.4 (3)°]. In the crystal, mol­ecules form [001] chains *via* weak non-classical C—H⋯N hydrogen bonds. The chains are stacked along the *b* axis.

## Related literature

For general background to the synthesis, chemical properties and probable applications in medicine (including computer program prognosis) of the title compound, see: Plati & Wenner (1949 ▶); Ellefson *et al.* (1978 ▶); Soldatenkov *et al.* (2009 ▶). For related compounds, see: Plati & Wenner (1950 ▶); Soldatenkov *et al.* (2008 ▶); Soldatova *et al.* (2010 ▶).
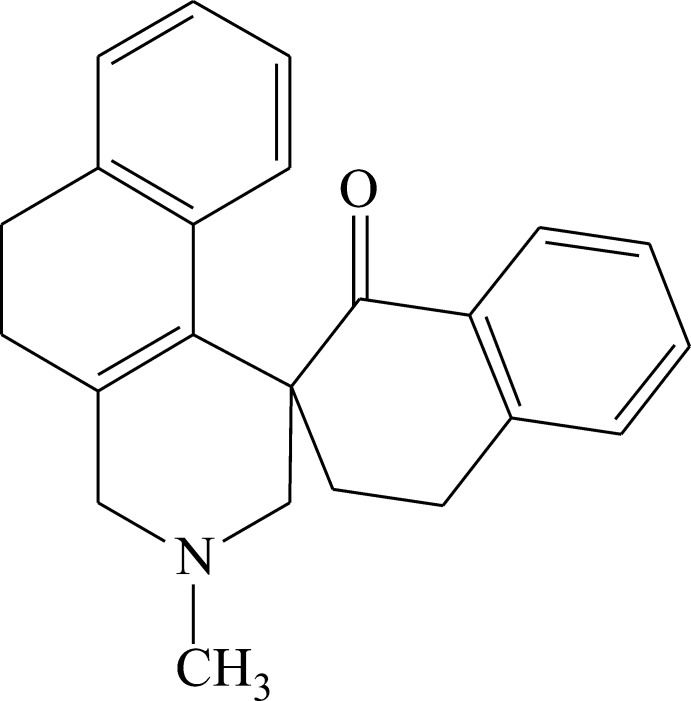


## Experimental

### 

#### Crystal data

C_23_H_23_NO
*M* *_r_* = 329.42
Monoclinic, 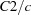

*a* = 27.645 (6) Å
*b* = 8.1613 (15) Å
*c* = 16.741 (3) Å
β = 116.037 (5)°
*V* = 3393.8 (11) Å^3^
*Z* = 8
Mo *K*α radiation
μ = 0.08 mm^−1^
*T* = 100 K
0.25 × 0.20 × 0.18 mm

#### Data collection

Bruker APEXII CCD diffractometer
Absorption correction: multi-scan (*SADABS*; Sheldrick, 2003 ▶) *T*_min_ = 0.981, *T*_max_ = 0.986
21756 measured reflections
4065 independent reflections
3080 reflections with *I* > 2σ(*I*)
*R*_int_ = 0.048

#### Refinement

*R*[*F* ^2^ > 2σ(*F* ^2^)] = 0.045
*wR*(*F* ^2^) = 0.122
*S* = 1.00
4065 reflections
227 parameters
H-atom parameters constrained
Δρ_max_ = 0.34 e Å^−3^
Δρ_min_ = −0.21 e Å^−3^

### 

Data collection: *APEX2* (Bruker, 2005 ▶); cell refinement: *SAINT* (Bruker, 2001 ▶); data reduction: *SAINT*; program(s) used to solve structure: *SHELXTL* (Sheldrick, 2008 ▶); program(s) used to refine structure: *SHELXTL*; molecular graphics: *SHELXTL*; software used to prepare material for publication: *SHELXTL*.

## Supplementary Material

Crystal structure: contains datablock(s) global, I. DOI: 10.1107/S1600536812043309/rk2383sup1.cif

Structure factors: contains datablock(s) I. DOI: 10.1107/S1600536812043309/rk2383Isup2.hkl

Additional supplementary materials:  crystallographic information; 3D view; checkCIF report

## Figures and Tables

**Table 1:** Hydrogen-bond geometry (Å, °)

*D*—H⋯*A*	*D*—H	H⋯*A*	*D*⋯*A*	*D*—H⋯*A*
C9—H9⋯N3^i^	0.95	2.59	3.534 (2)	171

Symmetry code: (i) 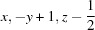.

**Fig. 1.**
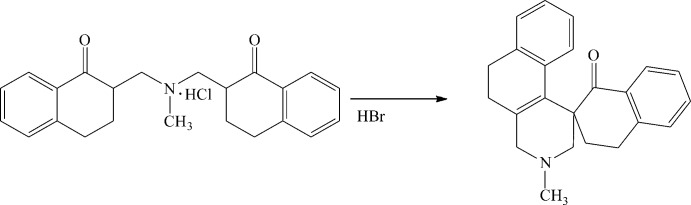
The preparation of the title product by a tandem transformation of bis(1–oxo–1,2,3,4–tertrahydro–2–naphthoylmethyl)amine hydrochloride.

**Fig. 2.**
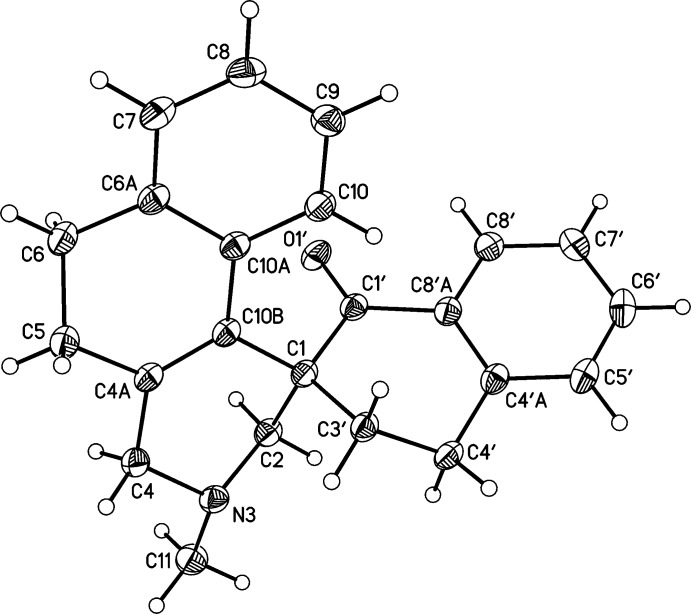
Molecular structure of I with the atom numbering scheme. Displacement ellipsoids are shown at the 50% probability level. H atoms are presented as a small spheres of arbitrary radius.

**Fig. 3.**
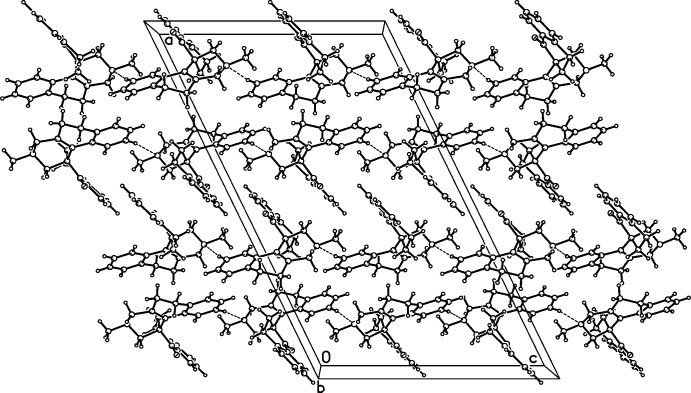
The H–bonded chains of I along the c axis. Dashed lines indicate the intermolecular hydrogen bonding interactions.

## supplementary crystallographic information

### Comment

The double Mannich bases, obtained in the form of hydrochlorides from acetophenones, formaldehyde and alkylamines by heating in HCl solution, can be easily cyclized under action of bases yielding 3–aroyl–4–arylpiperidin–4–ols (Plati & Wenner, 1949). The latter are intermediate products in the synthesis of important antihistaminic agents (Plati & Wenner, 1950; Ellefson *et al.*, 1978). We have synthesized an analogous double Mannich base - bis(1–oxo–1,2,3,4–tertrahydro–2–naphthoylmethyl)amine hydrochloride from α–tetralone and tried to prepare from it the corresponding γ–piperidol derivative by the same way. But, instead, multicomponent mixture was formed which contained only traces of the desirable derivative (as identified by LC—MS method). However, we have found that the expected product of the cyclization in the dehydrated form (Plati & Wenner, 1950; Soldatenkov *et al*., 2008, 2009; Soldatova *et al.*, 2010) is formed by heating of our double Mannich base in HBr solution (Fig. 1). It can be suggested that the starting reagent undergoes a tandem transformation. The first step of this process is aldol–type intramolecular cycloaddition of the two cyclohexenone moieties to each other, and the second one is dehydration. The structure of the product - spiro–*N*–methylhexahydrobenzo[*f*]isoquinoline–1,2'–(tetrahydronaphthalin–1'–one), C_23_H_23_NO, (I) was unambiguously established by X–ray diffraction study.

The molecule of I comprises spiro–fused hexahydrobenzo[*f*]isoquinoline and tetrahydronaphthalinone systems (Fig. 2). The tetrahydropyridine ring has a nonsymmetrical *half–chair* conformation (the C2 and N3 atoms are out of the plane through the other atoms of the ring by 0.612 (3)Å and -0.136 (3)Å, respectively), whereas the cyclohexadiene and cyclohexene rings adopt nonsymmetrical *half–boat* conformations (the C4A and C5 carbon atoms are out of the plane through the other atoms of the ring by 0.423 (3) and 0.814 (3) Å, respectively, in the case of the cyclohexadiene ring, and the C1 and C3' carbon atoms are out of the plane through the other atoms of the ring by 0.232 (3)Å and 0.756 (3)Å, respectively, in the case of the cyclohexene ring). The dihedral angle between the planes of the terminal benzene rings is 62.85 (6)°. The nitrogen N3 atom has a trigonal–pyramidal geometry (sum of the bond angles is 332.4 (3)°).

In the crystal, the molecules of I form the chains toward [0 0 1] by the weak non–classical intermolecular C9–H9···N3^i^ hydrogen bonding interactions (Fig. 3, Table 1). The crystal packing of the chains is stacking along the *b* axis. Symmetry code: (i) *x*, 1-*y*, -1/2+*z*.

The molecule of I possesses an asymmetric center at the C1 carbon atom. The crystal of I is racemate.

### Experimental

A solution of bis(1–oxo–1,2,3,4–tertrahydro–2–naphthoylmethyl)amine hydrochloride (2.31 g, 6.0 mmol) in 48% HBr (30 ml) was boiled for 2 h. The reaction mixture was cooled, poured into cold water (200 ml) and stirred at 293 K for 15 h. Then the pH of the mixture was brought upto 9, and the expected product was extracted by ether. The obtained extract was washed with water (50 ml) and dried over disodium sulfate. After the solvent evaporation, the residue was purified on the chromatographic column filled with alumogel (ether–hexane mixture, 1:1 as eluent). The main separated fraction (monitoring by *TLC*) was recrystallized from ethanol to give 0.72 g of yellow crystals of I. Yield is 24%. M.P. = 432–434 K. IR (KBr), ν/cm^-1^: 1673. ^1^H NMR (CDCl_3_, 400 MHz, 300 K): δ = 2.16–2.27 (m, 3H, C—CH_2_), 2.36 (s, 3H, CH_3_), 2.72–2.94 (m, 4H, C—CH_2_), 2.86 (s, 2H, NCH_2_), 3.07–3.28 (m, 3H, NCH_2_ and C—CH_2_), 6.74, 6.93, 7.01 and 7.10 (ABCD–system spectrum, 1H for each signal, ^3^J = 7.4, 7.2 and 7.1, H_arom_), 7.29, 7.36, 7.53 and 8.16 (ABCD–system spectrum, 1H for each signal, ^3^J = 7.7, 7.6 and 7.1, H_arom_). Mass spectrum (70 eV), *m/z* (I, %): 329 [*M*^+^] (97.3), 328 (14.1), 286 (21.7), 285 (40.2), 184 (19.7), 183 (22.1), 182 (18.8), 170 (100), 165 (22.5), 141 (38.3), 128 (23.1),115 (31.9), 91 (20.0), 90 (21.6). Anal. Calcd for C_23_H_23_NO: C, 83.85; H, 7.04; N, 4.25. Found: C, 83.92; H, 7.21; N, 4.36.

### Refinement

The hydrogen atoms were placed in calculated positions with C—H = 0.95Å–0.99Å and refined in the riding model with fixed isotropic displacement parameters (*U*_iso_(H) = 1.5*U*_eq_(C) for the methyl group and 1.2*U*_eq_(C) for the other groups).

### Crystal data

**Table:**

C_23_H_23_NO	*F*(000) = 1408
*M**_r_* = 329.42	*D*_x_ = 1.289 Mg m^−^^3^
Monoclinic, *C*2/*c*	Melting point = 432–434 K
Hall symbol: -C 2yc	Mo *K*α radiation, λ = 0.71073 Å
*a* = 27.645 (6) Å	Cell parameters from 4458 reflections
*b* = 8.1613 (15) Å	θ = 2.5–27.6°
*c* = 16.741 (3) Å	µ = 0.08 mm^−^^1^
β = 116.037 (5)°	*T* = 100 K
*V* = 3393.8 (11) Å^3^	Prism, yellow
*Z* = 8	0.25 × 0.20 × 0.18 mm

### Data collection

**Table:**

Bruker APEXII CCD diffractometer	4065 independent reflections
Radiation source: fine–focus sealed tube	3080 reflections with *I* > 2σ(*I*)
Graphite monochromator	*R*_int_ = 0.048
φ– and ω–scans	θ_max_ = 28.0°, θ_min_ = 2.5°
Absorption correction: multi-scan (*SADABS*; Sheldrick, 2003)	*h* = −36→36
*T*_min_ = 0.981, *T*_max_ = 0.986	*k* = −10→10
21756 measured reflections	*l* = −22→22

### Refinement

**Table:**

Refinement on *F*^2^	Primary atom site location: structure-invariant direct methods
Least-squares matrix: full	Secondary atom site location: difference Fourier map
*R*[*F*^2^ > 2σ(*F*^2^)] = 0.045	Hydrogen site location: inferred from neighbouring sites
*wR*(*F*^2^) = 0.122	H-atom parameters constrained
*S* = 1.00	*w* = 1/[σ^2^(*F*_o_^2^) + (0.0573*P*)^2^ + 3.1*P*] where *P* = (*F*_o_^2^ + 2*F*_c_^2^)/3
4065 reflections	(Δ/σ)_max_ < 0.001
227 parameters	Δρ_max_ = 0.34 e Å^−^^3^
0 restraints	Δρ_min_ = −0.21 e Å^−^^3^

### Special details

**Table:**

Geometry. All s.u.'s (except the s.u. in the dihedral angle between two l.s. planes) are estimated using the full covariance matrix. The cell s.u.'s are taken into account individually in the estimation of s.u.'s in distances, angles and torsion angles; correlations between s.u.'s in cell parameters are only used when they are defined by crystal symmetry. An approximate (isotropic) treatment of cell s.u.'s is used for estimating s.u.'s involving l.s. planes.
Refinement. Refinement of *F*^2^ against ALL reflections. The weighted *R*–factor *wR* and goodness of fit *S* are based on *F*^2^, conventional *R*–factors *R* are based on *F*, with *F* set to zero for negative *F*^2^. The threshold expression of *F*^2^ > σ(*F*^2^) is used only for calculating *R*–factors(gt) *etc*. and is not relevant to the choice of reflections for refinement. *R*–factors based on *F*^2^ are statistically about twice as large as those based on *F*, and *R*–factors based on ALL data will be even larger.

### Fractional atomic coordinates and isotropic or equivalent isotropic displacement parameters (Å^2^)

**Table:**

	*x*	*y*	*z*	*U*_iso_*/*U*_eq_	
C1	0.38819 (6)	0.38415 (17)	0.63564 (9)	0.0186 (3)	
C2	0.41181 (6)	0.31889 (18)	0.73225 (9)	0.0209 (3)	
H2A	0.4336	0.2196	0.7375	0.025*	
H2B	0.4357	0.4029	0.7734	0.025*	
N3	0.36865 (5)	0.27950 (15)	0.75652 (8)	0.0218 (3)	
C4	0.33687 (6)	0.14322 (18)	0.70326 (10)	0.0221 (3)	
H4A	0.3016	0.1428	0.7054	0.027*	
H4B	0.3555	0.0396	0.7304	0.027*	
C4A	0.32718 (6)	0.14692 (17)	0.60802 (9)	0.0195 (3)	
C5	0.28684 (6)	0.02231 (18)	0.54966 (10)	0.0228 (3)	
H5A	0.2504	0.0713	0.5236	0.027*	
H5B	0.2867	−0.0731	0.5861	0.027*	
C6	0.30040 (6)	−0.03436 (18)	0.47565 (10)	0.0233 (3)	
H6A	0.3335	−0.1018	0.5009	0.028*	
H6B	0.2707	−0.1030	0.4332	0.028*	
C6A	0.30877 (6)	0.11093 (18)	0.42766 (10)	0.0210 (3)	
C7	0.29174 (6)	0.10844 (19)	0.33643 (10)	0.0247 (3)	
H7	0.2731	0.0152	0.3032	0.030*	
C8	0.30145 (6)	0.2397 (2)	0.29267 (10)	0.0257 (3)	
H8	0.2895	0.2361	0.2301	0.031*	
C9	0.32863 (6)	0.37597 (19)	0.34078 (10)	0.0245 (3)	
H9	0.3358	0.4659	0.3115	0.029*	
C10	0.34541 (6)	0.38049 (18)	0.43231 (10)	0.0220 (3)	
H10	0.3639	0.4745	0.4648	0.026*	
C10A	0.33571 (6)	0.24990 (18)	0.47763 (9)	0.0190 (3)	
C10B	0.35079 (6)	0.25256 (17)	0.57463 (9)	0.0187 (3)	
C11	0.38899 (7)	0.2413 (2)	0.85111 (10)	0.0274 (3)	
H11A	0.3587	0.2200	0.8651	0.041*	
H11B	0.4099	0.3344	0.8865	0.041*	
H11C	0.4121	0.1441	0.8652	0.041*	
O1'	0.46093 (4)	0.29448 (13)	0.60614 (7)	0.0251 (3)	
C1'	0.43732 (6)	0.41176 (18)	0.61768 (9)	0.0191 (3)	
C3'	0.35611 (6)	0.54404 (17)	0.62726 (10)	0.0203 (3)	
H3A	0.3351	0.5709	0.5635	0.024*	
H3B	0.3304	0.5258	0.6529	0.024*	
C4'	0.39224 (6)	0.68881 (18)	0.67422 (10)	0.0216 (3)	
H4C	0.3699	0.7886	0.6641	0.026*	
H4D	0.4104	0.6676	0.7390	0.026*	
C4'A	0.43388 (6)	0.71699 (18)	0.64079 (10)	0.0210 (3)	
C5'	0.45125 (6)	0.87444 (19)	0.63298 (11)	0.0259 (3)	
H5C	0.4363	0.9666	0.6487	0.031*	
C6'	0.48995 (7)	0.89823 (19)	0.60268 (11)	0.0286 (4)	
H6C	0.5008	1.0063	0.5969	0.034*	
C7'	0.51303 (6)	0.7651 (2)	0.58076 (10)	0.0263 (3)	
H7A	0.5400	0.7814	0.5609	0.032*	
C8'	0.49630 (6)	0.60865 (19)	0.58814 (10)	0.0225 (3)	
H8A	0.5122	0.5172	0.5736	0.027*	
C8'A	0.45636 (6)	0.58295 (18)	0.61671 (9)	0.0201 (3)	

### Atomic displacement parameters (Å^2^)

**Table:**

	*U*^11^	*U*^22^	*U*^33^	*U*^12^	*U*^13^	*U*^23^
C1	0.0219 (7)	0.0156 (7)	0.0175 (7)	0.0003 (5)	0.0080 (6)	−0.0009 (5)
C2	0.0237 (7)	0.0184 (7)	0.0182 (7)	0.0011 (6)	0.0071 (6)	−0.0007 (5)
N3	0.0268 (6)	0.0213 (6)	0.0169 (6)	−0.0007 (5)	0.0092 (5)	−0.0014 (5)
C4	0.0277 (8)	0.0184 (7)	0.0220 (7)	0.0002 (6)	0.0126 (6)	0.0007 (6)
C4A	0.0216 (7)	0.0162 (7)	0.0199 (7)	0.0018 (5)	0.0082 (6)	−0.0016 (5)
C5	0.0249 (7)	0.0187 (7)	0.0236 (8)	−0.0014 (6)	0.0095 (6)	−0.0005 (6)
C6	0.0266 (8)	0.0173 (7)	0.0233 (7)	−0.0011 (6)	0.0086 (6)	−0.0024 (6)
C6A	0.0230 (7)	0.0179 (7)	0.0209 (7)	0.0031 (5)	0.0085 (6)	−0.0015 (6)
C7	0.0275 (8)	0.0219 (7)	0.0217 (7)	0.0011 (6)	0.0081 (6)	−0.0051 (6)
C8	0.0309 (8)	0.0277 (8)	0.0181 (7)	0.0039 (6)	0.0105 (6)	−0.0010 (6)
C9	0.0297 (8)	0.0233 (8)	0.0217 (7)	0.0023 (6)	0.0125 (6)	0.0024 (6)
C10	0.0254 (7)	0.0191 (7)	0.0213 (7)	0.0003 (6)	0.0100 (6)	−0.0021 (6)
C10A	0.0198 (7)	0.0180 (7)	0.0185 (7)	0.0023 (5)	0.0077 (6)	−0.0010 (5)
C10B	0.0202 (7)	0.0161 (6)	0.0183 (7)	0.0018 (5)	0.0069 (6)	−0.0011 (5)
C11	0.0352 (9)	0.0263 (8)	0.0197 (8)	0.0007 (7)	0.0113 (7)	0.0015 (6)
O1'	0.0272 (6)	0.0189 (5)	0.0299 (6)	0.0037 (4)	0.0132 (5)	−0.0033 (4)
C1'	0.0213 (7)	0.0184 (7)	0.0153 (7)	0.0013 (5)	0.0060 (5)	−0.0005 (5)
C3'	0.0231 (7)	0.0164 (7)	0.0212 (7)	0.0017 (5)	0.0095 (6)	−0.0004 (5)
C4'	0.0261 (7)	0.0166 (7)	0.0220 (7)	0.0023 (6)	0.0105 (6)	−0.0030 (6)
C4'A	0.0219 (7)	0.0185 (7)	0.0184 (7)	0.0007 (5)	0.0049 (6)	−0.0011 (5)
C5'	0.0279 (8)	0.0178 (7)	0.0288 (8)	0.0009 (6)	0.0094 (7)	−0.0018 (6)
C6'	0.0294 (8)	0.0197 (7)	0.0325 (9)	−0.0036 (6)	0.0097 (7)	0.0028 (6)
C7'	0.0259 (8)	0.0276 (8)	0.0255 (8)	−0.0010 (6)	0.0115 (6)	0.0042 (6)
C8'	0.0246 (7)	0.0215 (7)	0.0193 (7)	0.0035 (6)	0.0077 (6)	0.0017 (6)
C8'A	0.0218 (7)	0.0188 (7)	0.0170 (7)	0.0001 (5)	0.0060 (6)	−0.0001 (5)

### Geometric parameters (Å, º)

**Table:**

C1—C10B	1.5292 (19)	C9—C10	1.393 (2)
C1—C1'	1.531 (2)	C9—H9	0.9500
C1—C2	1.549 (2)	C10—C10A	1.401 (2)
C1—C3'	1.5491 (19)	C10—H10	0.9500
C2—N3	1.4554 (19)	C10A—C10B	1.4889 (19)
C2—H2A	0.9900	C11—H11A	0.9800
C2—H2B	0.9900	C11—H11B	0.9800
N3—C4	1.4548 (19)	C11—H11C	0.9800
N3—C11	1.4626 (19)	O1'—C1'	1.2207 (17)
C4—C4A	1.497 (2)	C1'—C8'A	1.495 (2)
C4—H4A	0.9900	C3'—C4'	1.524 (2)
C4—H4B	0.9900	C3'—H3A	0.9900
C4A—C10B	1.343 (2)	C3'—H3B	0.9900
C4A—C5	1.509 (2)	C4'—C4'A	1.502 (2)
C5—C6	1.516 (2)	C4'—H4C	0.9900
C5—H5A	0.9900	C4'—H4D	0.9900
C5—H5B	0.9900	C4'A—C5'	1.398 (2)
C6—C6A	1.506 (2)	C4'A—C8'A	1.402 (2)
C6—H6A	0.9900	C5'—C6'	1.384 (2)
C6—H6B	0.9900	C5'—H5C	0.9500
C6A—C7	1.387 (2)	C6'—C7'	1.389 (2)
C6A—C10A	1.412 (2)	C6'—H6C	0.9500
C7—C8	1.389 (2)	C7'—C8'	1.382 (2)
C7—H7	0.9500	C7'—H7A	0.9500
C8—C9	1.385 (2)	C8'—C8'A	1.398 (2)
C8—H8	0.9500	C8'—H8A	0.9500
			
C10B—C1—C1'	111.72 (11)	C9—C10—C10A	121.69 (14)
C10B—C1—C2	107.91 (11)	C9—C10—H10	119.2
C1'—C1—C2	104.59 (11)	C10A—C10—H10	119.2
C10B—C1—C3'	109.80 (11)	C10—C10A—C6A	117.82 (13)
C1'—C1—C3'	112.35 (12)	C10—C10A—C10B	123.45 (13)
C2—C1—C3'	110.26 (11)	C6A—C10A—C10B	118.70 (13)
N3—C2—C1	110.24 (12)	C4A—C10B—C10A	119.36 (13)
N3—C2—H2A	109.6	C4A—C10B—C1	118.86 (13)
C1—C2—H2A	109.6	C10A—C10B—C1	121.48 (12)
N3—C2—H2B	109.6	N3—C11—H11A	109.5
C1—C2—H2B	109.6	N3—C11—H11B	109.5
H2A—C2—H2B	108.1	H11A—C11—H11B	109.5
C4—N3—C2	110.27 (11)	N3—C11—H11C	109.5
C4—N3—C11	110.03 (12)	H11A—C11—H11C	109.5
C2—N3—C11	112.12 (12)	H11B—C11—H11C	109.5
N3—C4—C4A	114.52 (12)	O1'—C1'—C8'A	121.05 (13)
N3—C4—H4A	108.6	O1'—C1'—C1	119.84 (13)
C4A—C4—H4A	108.6	C8'A—C1'—C1	119.10 (12)
N3—C4—H4B	108.6	C4'—C3'—C1	112.75 (12)
C4A—C4—H4B	108.6	C4'—C3'—H3A	109.0
H4A—C4—H4B	107.6	C1—C3'—H3A	109.0
C10B—C4A—C4	124.35 (13)	C4'—C3'—H3B	109.0
C10B—C4A—C5	121.30 (13)	C1—C3'—H3B	109.0
C4—C4A—C5	114.34 (12)	H3A—C3'—H3B	107.8
C4A—C5—C6	110.93 (12)	C4'A—C4'—C3'	111.25 (12)
C4A—C5—H5A	109.5	C4'A—C4'—H4C	109.4
C6—C5—H5A	109.5	C3'—C4'—H4C	109.4
C4A—C5—H5B	109.5	C4'A—C4'—H4D	109.4
C6—C5—H5B	109.5	C3'—C4'—H4D	109.4
H5A—C5—H5B	108.0	H4C—C4'—H4D	108.0
C6A—C6—C5	110.29 (12)	C5'—C4'A—C8'A	118.47 (14)
C6A—C6—H6A	109.6	C5'—C4'A—C4'	121.76 (13)
C5—C6—H6A	109.6	C8'A—C4'A—C4'	119.77 (13)
C6A—C6—H6B	109.6	C6'—C5'—C4'A	121.04 (14)
C5—C6—H6B	109.6	C6'—C5'—H5C	119.5
H6A—C6—H6B	108.1	C4'A—C5'—H5C	119.5
C7—C6A—C10A	120.00 (14)	C5'—C6'—C7'	120.39 (14)
C7—C6A—C6	121.23 (13)	C5'—C6'—H6C	119.8
C10A—C6A—C6	118.75 (13)	C7'—C6'—H6C	119.8
C6A—C7—C8	121.25 (14)	C8'—C7'—C6'	119.26 (14)
C6A—C7—H7	119.4	C8'—C7'—H7A	120.4
C8—C7—H7	119.4	C6'—C7'—H7A	120.4
C9—C8—C7	119.58 (14)	C7'—C8'—C8'A	120.98 (14)
C9—C8—H8	120.2	C7'—C8'—H8A	119.5
C7—C8—H8	120.2	C8'A—C8'—H8A	119.5
C8—C9—C10	119.64 (14)	C8'—C8'A—C4'A	119.83 (14)
C8—C9—H9	120.2	C8'—C8'A—C1'	118.57 (13)
C10—C9—H9	120.2	C4'A—C8'A—C1'	121.56 (13)
			
C10B—C1—C2—N3	−58.09 (15)	C1'—C1—C10B—C4A	141.08 (13)
C1'—C1—C2—N3	−177.18 (11)	C2—C1—C10B—C4A	26.64 (17)
C3'—C1—C2—N3	61.83 (15)	C3'—C1—C10B—C4A	−93.57 (15)
C1—C2—N3—C4	65.76 (15)	C1'—C1—C10B—C10A	−45.28 (17)
C1—C2—N3—C11	−171.26 (12)	C2—C1—C10B—C10A	−159.72 (12)
C2—N3—C4—C4A	−39.30 (17)	C3'—C1—C10B—C10A	80.07 (16)
C11—N3—C4—C4A	−163.49 (13)	C10B—C1—C1'—O1'	−43.04 (18)
N3—C4—C4A—C10B	8.3 (2)	C2—C1—C1'—O1'	73.43 (16)
N3—C4—C4A—C5	−170.33 (12)	C3'—C1—C1'—O1'	−166.97 (13)
C10B—C4A—C5—C6	32.90 (19)	C10B—C1—C1'—C8'A	138.38 (13)
C4—C4A—C5—C6	−148.37 (13)	C2—C1—C1'—C8'A	−105.15 (14)
C4A—C5—C6—C6A	−50.93 (16)	C3'—C1—C1'—C8'A	14.45 (17)
C5—C6—C6A—C7	−142.78 (14)	C10B—C1—C3'—C4'	−170.57 (12)
C5—C6—C6A—C10A	38.82 (18)	C1'—C1—C3'—C4'	−45.58 (16)
C10A—C6A—C7—C8	1.1 (2)	C2—C1—C3'—C4'	70.66 (15)
C6—C6A—C7—C8	−177.28 (14)	C1—C3'—C4'—C4'A	56.09 (16)
C6A—C7—C8—C9	0.0 (2)	C3'—C4'—C4'A—C5'	144.85 (14)
C7—C8—C9—C10	−0.7 (2)	C3'—C4'—C4'A—C8'A	−35.28 (19)
C8—C9—C10—C10A	0.3 (2)	C8'A—C4'A—C5'—C6'	−0.4 (2)
C9—C10—C10A—C6A	0.8 (2)	C4'—C4'A—C5'—C6'	179.52 (14)
C9—C10—C10A—C10B	−177.23 (14)	C4'A—C5'—C6'—C7'	−1.1 (2)
C7—C6A—C10A—C10	−1.5 (2)	C5'—C6'—C7'—C8'	1.0 (2)
C6—C6A—C10A—C10	176.93 (13)	C6'—C7'—C8'—C8'A	0.4 (2)
C7—C6A—C10A—C10B	176.67 (13)	C7'—C8'—C8'A—C4'A	−1.9 (2)
C6—C6A—C10A—C10B	−4.9 (2)	C7'—C8'—C8'A—C1'	176.02 (13)
C4—C4A—C10B—C10A	−176.81 (13)	C5'—C4'A—C8'A—C8'	1.8 (2)
C5—C4A—C10B—C10A	1.8 (2)	C4'—C4'A—C8'A—C8'	−178.06 (13)
C4—C4A—C10B—C1	−3.0 (2)	C5'—C4'A—C8'A—C1'	−176.02 (13)
C5—C4A—C10B—C1	175.55 (13)	C4'—C4'A—C8'A—C1'	4.1 (2)
C10—C10A—C10B—C4A	161.01 (14)	O1'—C1'—C8'A—C8'	10.3 (2)
C6A—C10A—C10B—C4A	−17.0 (2)	C1—C1'—C8'A—C8'	−171.18 (12)
C10—C10A—C10B—C1	−12.6 (2)	O1'—C1'—C8'A—C4'A	−171.88 (14)
C6A—C10A—C10B—C1	169.35 (13)	C1—C1'—C8'A—C4'A	6.7 (2)

### Hydrogen-bond geometry (Å, º)

**Table:**

*D*—H···*A*	*D*—H	H···*A*	*D*···*A*	*D*—H···*A*
C9—H9···N3^i^	0.95	2.59	3.534 (2)	171

Symmetry code: (i) *x*, −*y*+1, *z*−1/2.
